# Hospital Practice

**Published:** 1828-04-01

**Authors:** 


### HOSPITAL PRACTICE.

### 1. LA CHARITE.

REMARKABLE DISEASE OF THE MUCOUS MEMBRANE OF THE BLADDER. By M. LOUIS.

This distinguished pathologist remarks that, of all other mucous membranes, that of the bladder is the least frequently found diseased. This is the more to be wondered at, when we consider the great variety of alterations to which the urine is subject, from diseases and from diet. M. Louis examined the mucous membrane of the bladder in 500 subjects, dead of various diseases, and found six instances where this surface was affected with simple redness or injection of vessels, but without any softening of structure. In two or three other cases, there were softening and other species of organic lesion. Two cases we shall present in an abridged form to our readers.

*Case* 1. A man, aged 77 years, was brought to the hospital, on the 23d March, 1827, in a desperate condition. It was learnt that, lately, he had been obliged to make water very frequently, but without pain. Two or three times he passed blood in his urine in considerable quantities—and, for a fortnight before he entered the hospital, his water contained more or less blood. He died the next night, presenting gangrene of the lower extremities, &c.

Passing over the morbid appearances in other parts of the body, we find that the bladder was contracted to about the size of a man's fist, and was slightly prominent above the pubes, being adherent to the transverse arch of the colon by a membranous cord, of about two inches in length. It contained three or four ounces of dark-coloured, purulent liquid. The internal surface of this receptacle was red, and presented an adventitious tissue, better than a line in thickness, and of a filamentous structure, but quite soft and easily lacerable. The urethra was perfectly sound. M. Louis has not been able to find a case of morbid anatomy of the bladder, precisely similar to the above, in any of our pathological writers. There can be no doubt, however, that it was a product of inflammation—and, moreover, that it was rather a morbid development of the mucous membrane itself than a new or adventitious structure formed by disease.

This patient died of a rupture of the left auricle of the heart, within the pericardium—an extremely rare accident.

*Case* 2. A female, aged 45 years, was received into LA CHARITÉ, on the 15th March, 1827, stating that she had been ill about eight months. In the beginning of that period, she had some uterine discharges, accompanied by pains in the hypogastrium and loins, which she characterized as lancinating and tearing, and with hardly any remissions.

The right thigh had lately become the seat of a disagreeable sensation of formication. The urine was passed very frequently, and in small quantities at a time, during the preceding four months. The bowels were constipated—the disease had been left to itself. On examination, there was perceived a swelling in the hypogastrium, rising a couple of inches above the pubes, and accompanied by the most acute sensibility on pressure. There was some inconsiderable discharge from the vagina. No fever; but the vital powers were evidently sinking. She lingered out till the 28th of April, when she died. Latterly she had been affected with diarrhœa.

*Dissection.* Omitting the notice of some unimportant lesions in the stomach and bowels, we come to the urinary organs. The kidneys were pale, and not more than half their natural size, and yet their pelves and infundibula were very much enlarged. The lining membrane of these last parts, as well as that of the ureters, was thrice its natural thickness. The bladder was very small. The internal surface presented a strange medley of morbid productions, which it would be very difficult to describe. There were three layers, as it were, of diseased growths—one appeared to consist of pyriform vesicles, demi-transparent, containing a clear, but yellowish fluid. These were mixed with, or joined to, another set of bodies, bearing more the charater of tubercular bodies. The mucous membrane, and the submucous tissue, was in a state of great disease. The uterus presented several scirrhous masses growing about its cervix and body.—REPERTOIRE.

The above are curious specimens of morbid anatomy.

We have said that rupture of the left auricle of the heart, within the pericardium, is a rare accident. We lately saw, in the possession of Dr. Somerville, jun. a fine specimen of aneurismal pouch, of aortic origin, but within the pericardium —that is, behind the semilunar valves. It had attained the size of a large goose-egg—and, ultimately, burst into the cavity of the pericardium, causing immediate death, of course.

---

### 2. HOPITAL DES ENFANS MALADES.

#### PERIPNEUMONY OF CHILDREN.

In the HÔPITAL DES ENFANS MALADES, M. Guersent, has lately been induced to try the efficacy of tartarized antimony, in the pulmonic inflammations of children, in the above institution, and has published, in the September Number of the ARCHIVES GENERALES, a few cases illustrative of this plan of treatment M. Guersent, does not, like Laennec, and some of the Italian physicians, trust, almost exclusively, to antimony. He bleeds vigorously at first—but finding that this measure is insufficient, he employs the above remedy in large doses. Thus, in the first case, a boy of 12 years of age—the inflammation occupied almost the whole of the left lung, and the second stage, or hepatization, had taken place. The patient had been repeatedly bled and leeched, but still the cough, the dyspnœa, the fever, the viscid and scanty expectoration, threatened the boy's life. In this state, M. Guersent ordered six grains of tartar emetic to be dissolved in twelve ounces of orange-flower water, and this portion to be taken in divided doses, every two or three hours, in the course of one day and night. The first dose vomited the child once, and produced three or four alvine evacuations. The three next doses occasioned neither sickness nor purging. The little patient had some sleep, and next morning the symptoms were mitigated. During the succeeding two days, the same medicine was administered, without any inconvenience, though the daily quantum was augmented to eight grains. The disease was very quickly subdued. Some of the other cases were more formidable, and the same treatment was adopted with success.—ARCHIVES.

---

### 3. MEATH HOSPITAL, DUBLIN.

#### HEPATIC DISEASE. DRS. GRAVES and STOKES.

In the following case, related by Drs. Graves and Stokes, from the Meath Hospital, some observations will be found, corroborating certain opinions which we gave in our review of Dr. Bright's work, in the second Fasciculus of this Number, page 319.

*Case.* J. M'Clusky, aged 11 years, of scrofulous habit, labours under considerable swelling of the abdomen, with fluctuation and tympanitis. "The right hypochondrium appears rather full; upon examination, the liver can be felt extending across the left hypochondrium, and as far down as the umbilicus, presenting a defined edge." He had œdema of the left leg—no cough or dyspnœa—good appetite—much thirst—urine copious, light-coloured—and depositing albumen in abundance when heated. Bowels are regular, the pulse 125, tongue clean and moist, sleeps well. The complaint was of twelve months standing. One grain of calomel was ordered to be taken every night, and some mercurial frictions to the right hypochondrium. On the eighth day of this treatment, the mouth was affected, and the calomel was omitted. Having caught cold, a smart ophthalmia occurred, which required leeching, &c. Afterwards, as the swelling of the abdomen continued, the calomel was renewed, in two grain doses, and half a drachm of the spir. æth. nitr. was given twice a day. The belly became reduced in size—the urine more copious, but still coagulable by heat. In a short time afterwards, he was discharged, the abdomen being nearly reduced to its natural size, and the appetite good. The following extract from the observations appended to this case, we deem worthy of insertion.

"It is not easy to determine the nature of the hepatic tumour which was so very considerable in this boy. It was slow in its increase, and not attended with well marked symptoms of chronic inflammation of the liver. There was no tenderness on pressure, nor any pain or uneasiness in the right hypochondrium. There was no evident derangement of the biliary secretion, and his appetite, sleep, strength, and nutrition, were scarcely impaired. On the other hand, the œdema of the left leg, the commencing ascites and tympanitis, the albuminous urine, accelerated pulse and increased thirst, all united to prove that the constitution had begun to suffer in consequence of the diseased state of the liver. The alterative doses of mercury, which were cautiously exhibited, were evidently productive of much benefit. The mixture with spirit of nitrous æther seemed useful in relieving the Tympanitis. *In this, and many other cases, where considerable organic alteration had taken place in the liver, we have observed an apparently healthy secretion of bile. Thus we have found bile of an healthy colour and consistence in the gall bladder, when the substance of the liver was tuberculated throughout. Naturally coloured alvine discharges therefore furnish no proof that extensive organic disease of the liver does not exist.*"

"Concerning the albuminous state of the urine we may remark, that it is no proof of an inflammatory condition of the constitution, it merely indicates considerable disorder of the function of assimilation. In health, a certain portion of animal matter is contained in the urine, in the form of that highly animalized substance, urea. This may be increased so much in quantity above the healthy standard as to constitute a disease. When the assimilative powers are more deranged, the animal matter of the urine ceases to assume the more highly animalized form of urea, and is voided in the form of albumen, which contains much less nitrogen than urea. In a state of the system still further depraved, it passes off in the form of sugar, which contains no nitrogen, and is the least highly animalized. In diabetes, it is probable, that the urea is voided in increased quantity at first; as the disease proceeds the animal matter is voided in the shape of albumen, and afterwards of sugar. When diabetic patients are getting better, then the contrary seems to take place; and when the sugar diminishes the albumen increases or reappears, and afterwards is replaced by the more healthy secretion of urea. Doctor Prout was the first to establish the existence of these three different species, or rather stages of diabetes. In dropsy the appearance of albumen in the urine is a bad sign, as indicating a depraved assimilation and a source of debility. We have established, by numerous experiments, that when there is much albumen, there is scarcely any urea in the urine, and *vicê versâ*, or more generally that the proportion of urea is inversely as that of the albumen. How far the treatment suited to diabetes may be also applicable to cases of chronic dropsy with albuminous urine, and unattended by organic disease, experience alone can decide."

The passage which we have marked in italics, is that to which we wish to draw the attention of Dr. Bright, and the public. But the whole of the observations of our intelligent authors are deserving of notice.

### Pathology of Fever. Drs. Graves and Stokes.

We believe that none, even of our continental brethren, have taken greater pains to investigate the causes, the seat and the consequences of fever, than the two physicians above-named. Their sentiments, therefore, on the pathology of this wide spread disease must be interesting to the profession at large. These sentiments may be gathered from some observations appended to a case of fever (among many others) treated in the Meath Hospital.

"The epigastric tenderness, with nausea and vomiting, so common in this fever, seemed to be caused by inflammation of the mucous membrane of the stomach. In most of the fatal cases this was found of a dark-red colour and very soft, a condition evidently produced by violent inflammation. In others the redness was not so extensive, dark, or continuous; although we acknowledge, that in the present fever the above symptoms, depending on inflammation of the stomach, are very frequent, yet we have seen some cases where there was no evidence of any local inflammation whatsoever; and in others again, have observed that some other organ, as the *brain* or *lungs*, was the seat of inflammation, while the stomach was free. We cannot subscribe therefore to the opinion, which supposes a local inflammation to be the root of all fevers, or to that which attributes their origin solely to inflammation of the mucous membrane of the stomach and alimentary canal. In our dissections we have in some cases found the brain inflamed, in more cases the lungs, and still more some part of the digestive organs. We do not recollect to have found in one instance, out of very numerous dissections, a fatal case of fever which did not exhibit some serious local lesion of an inflammatory nature; so that while we deny, from our observation of cases during life, that fever necessarily implies local inflammation, dissection has convinced us that the occurrence of local inflammation during fever, is the general cause of its fatal termination. Let us here observe that the latter inference is by no means contradictory of the former; for in the fatal cases, accurate observation always detected, *during life*, the seat of the inflammation; so that in those cases which terminated favourably, and where no such symptoms existed, our inference that no local inflammation had been present, receives additional strength from our post mortem examinations, for there we always found inflammation where we expected to find it; that is to say, we were always able to pronounce on its situation, so far as to tell, before the body was opened, *in which cavity the inflammation would be found.* The post mortem examinations have been always conducted by ourselves with the greatest care, and we generally spend between two and three hours in the examination of the body, being convinced that nothing has contributed to retard the advancement of medicine so much as superficial post mortem examinations. In examining the abdomen, we first note the general appearances of the intestines, and then take out the whole intestinal tube, which we slit up with an *enterotome* at its mesenteric attachment; this is done in order to avoid dividing any of the follicular patches or ulcerations in the small intestines, which are always situated at some distance from the mesenteric attachment. During this process we examine the contents of the alimentary canal; and afterwards having first carefully washed the mucous membrane, we remark its appearance throughout its whole extent from the stomach to the rectum. The morbid anatomy of the brain, the lungs, and the intestinal canal, has, within these very few years, received so many important additions, so much light has been thrown on this subject by more accurate investigations, that we would hesitate much in drawing any conclusions from the dissections of fever subjects recorded before this period. Indeed, we could prove that in most of even modern works on the pathology of fever, morbid appearances have been frequently mistaken, and more especially that many things, both in the brain, lungs, and alimentary canal, have been set down as morbid, which really are not so; consequently, conclusions, not at all justified by the state of the parts, have been drawn. Thus, we hear of sanguineous congestion in the head, and morbid vascularity of the brain, intestines, &c. where the very accounts given contain internal evidence, that these supposed morbid appearances had either no claim to that appellation, or resulted from changes which took place immediately before or after death. In fact, we look upon the morbid anatomy of fever as a subject which requires to be investigated almost *de novo*. Our assertion, that we have hitherto found evident lesions of vital organs in all the fever subjects we have dissected, is, we are aware, opposed to the recorded experience of many authors, who relate numerous cases in which no morbid alteration of any consequence could be detected. We question however very much the accuracy of such dissections, for, as has been well observed by Rostan, nothing is easier than to find nothing. We doubt whether such persons have injured medical science more than those who have found too much."

We recommend the above careful mode of *post mortem* researches to the attention of our brethren in this country—especially those who have the superintendance of public institutions, and who, consequently, have less difficulties to contend with, than those in private practice. We believe that very few cases of fever go on to a fatal termination, without the occurrence of organic changes—at least of inflammation in some of the great viscera. These may be the cause of death, but not of the fever.

---

### 4. ROYAL INFIRMARY OF EDINBURGH.

In a paper on bronchotomy, Dr. Cullen has related the following interesting case that occurred some years ago, in the Royal Infirmary.

*Case.* A healthy married woman, aged 25 years, having laboured under considerable depression of spirits and mental distress, attempted suicide with a table-knife, but only made a flesh-wound over the crico-thyroid membrane, which was slightly perforated. Through this aperture the woman breathed, when Dr. Cullen saw her two hours afterwards. The wound was not of much consequence in itself, but the patient was in an unpleasant condition in other respects. She was slightly delirious—dwelling on her domestic grievances—pulse small and feeble—face flushed—tongue white and tremulous—countenance expressive of anxiety and suffering. The wound was dressed—the head shaved—and ice applied to the scalp. Some wine and water was given to her, when she was disposed to sink—a cathartic was administered, and she was confined in a dark room. She passed a quiet but sleepless night, and next day had cough. Delirium returned in the evening. Leeches to the temples and forehead. *Third day*. Rather better—cough, with difficult expectoration. In the evening, the stethoscope detected inflammation in the smaller branches of the bronchia. A large blister to the sternum. *Fourth day*. Delirious, probably from agitation at seeing her husband. *Fifth* morning, early, she was reported to be dying. Dr. Lubbock, the house-surgeon, found her on the point of suffocation, with death-rattle in the throat—livid face—cold extremities. Dr. L. quickly introduced a large curved canula into the wound, which enabled the patient to take a deep inspiration, and the breathing immediately became easier. She now brought up a quantity of mucus through the canula by a process which Dr. C. will not allow to be exactly coughing. The alarming symptoms soon subsided, and the expectoration became free. The same train of dangerous symptoms again occurred in the evening from the slipping out of the canula, but were again relieved by closing the opening in the larynx, and inducing the woman to cough up the mucus by the natural passage. This, however, had but a temporary effect, and she died the same night.

*Dissection*. The vessels of the brain were found much injected, with considerable effusion in the sub-arachnoid cellular tissue. There was no disorganization of the substance of the brain, but there was much serum in the ventricles and at the base of the brain. The bronchia, especially on the left side, were obstructed with bloody mucus—the lungs sound.

The mucous membrane, in the neighbourhood of the wound, was inflamed but not thickened. There was no effusion into the submucous cellular tissue, and, consequently, no diminution of diameter in the glottis. The membrane of the trachea and bronchia was highly inflamed. —ED. JOURNAL.

Dr. Cullen seems to think that this poor woman's death was occasioned by the state of the air-passages. But we imagine the phenomena presented in the brain were more likely to occasion the fatal event. The case, however, has been evidently brought forward to illustrate some observations made in a preceding number of our Northern contemporary, on the difficulty which a patient has in coughing up, or rather expectorating the phlegm, after bronchotomy. Indeed, it is probable that, had it not been for some observations which we made on Dr. Cullen's paper, this last case might not have seen the light. We said that a patient can cough—that is, forcibly expectorate through a tube in the trachea. Dr. C. maintains that is not coughing, strictly speaking. We will not quarrel with the learned Doctor about the name of process; but, having seen a patient cough for weeks and months through a canula, we cannot give up the evidence of our senses, even to support an ingenious hypothesis. If Dr. Cullen will look into the 5th volume of the Medico-Chirurgical Journal and Review, for January, 1818, he will there see the case of Mr. Price, of Portsmouth, who, to this day, breathes through the tube. Dr. C. will see the diurnal details of the case, where the act of coughing is every day stated—and where, on the 55th day after the operation, the following passage occurs:—"Last night he felt a piece of bone (ossification of the thyroid cartilage) fall down into the lungs, and has ever since been in a dreadful state of *coughing*. He feels the piece come up to the tube, but, as he cannot get it out, it falls back, and keeps him in constant agitation." A large canula was quickly constructed, and forced into the aperture, and, "shortly afterwards, in a *violent convulsive cough*, he threw out the piece of bone (No. 1) *quite across the room* where he was sitting." P. 6. Now we think Dr. Cullen will absolve us from the charge of forging a case ten years ago, and putting down expressions that were to contradict his theory at the present time. After this document, we leave him to persuade the world, *by words*, that a man cannot *cough* after a tube has been introduced into the trachea.

### 5. GLASGOW INFIRMARY—HOSPITAL OF SURGERY, &C.

### PULSATING TUMOURS OF THE SCALP.

The first case which we shall notice, occurred to Dr. Maclachlan, one of the surgeons to the Glasgow Royal Infirmary, and is related by that gentleman, in the first number of the Glasgow Medical Journal.

The patient, a discharged soldier, æt. 30, applied to Dr. M. with a tumour on the left side of the scalp, presenting the following appearances:

"Soft, puffy, pulsating, and somewhat elastic swellings of a varicose appearance were found to occupy the course of the temporal, posterior auris, and occipital arteries and their principal branches; each branch terminating by a tortuous extremity. These swellings could be made partly to disappear on pressure, but on its removal, they speedily regained their former volume. They pulsated throughout their whole extent, and the pulsations were synchronous with those of the heart. By pressing on the common carotid, the pulsations ceased all along the swellings; and, by intercepting the flow of blood through the temporal or posterior auris, the throb was interrupted in corresponding parts of the tumour. They were not painful on being handled, but he complained much of the torture he had experienced for the last two months, from the throbbing, which often deprived him of rest for nights together, and, as he said, made his existence miserable to him. The integuments covering the swellings were of their natural colour; only at those points which were most prominent, they had a slightly blueish-red tinge.

"This *arborescent* tumour commenced in front of the ear, immediately over the zygoma, and quickly swelling out, it became of the size of a split lemon, lying transversely over the ear. It sent a process forwards on the forehead, communicating by a tortuous extremity with the supra-orbitary twig from the internal carotid; a large process upwards to the crown of the head; and backwards, the main body of the tumour communicated with the puffy swellings of the posterior auris and occipitalis, which latter vessels gave a varicose feeling to the scalp over the left side of the occiput.

"The largest and most prominent part of the tumour was immediately over the ear: at this point, the throbbing was very violent, and the integuments being very thin and rather pointing, it threatened ere long to burst."

Ten years previously the temporal artery had been opened, and a small aneurism formed upon it. For this the vessel was cut across, but without success, and a ligature was then applied. The little tumour disappeared for a time, but afterwards returned, though for the first five years it gave him little uneasiness. Pressure had been already employed, and the patient would not again submit to it, but wished his carotid artery to be tied. Dr. M. however, proposed taking up the vessels separately that fed the tumour, and, if this should fail, tying the common carotid. Accordingly, assisted by Professor Towers and Dr. Anderson, he exposed the temporal artery, as it emerges from under the parotid, and found the vessel larger than a goose-quill, thinner and more diaphanous in its coats than a vein, and pulsating with much violence. A ligature was applied upon the vessel, with a compress and bandage for additional security. Pulsation ceased in the anterior and central parts of the tumour, which felt flaccid and doughy, but, Dr. M. not liking the state of the vessel, determined on securing the carotid, which was done next day in the presence of Professors Burns and Towers, Drs. King and Anderson. The steps of the operation, which was performed on the 10th July, we need not describe, but, immediately after its completion, the tumours of the head felt flaccid and lost their pulsation, although they were but little diminished in size. Next day he was seized with pain in the right side; pulse 120; skin hot. *V. S. at two bleedings, to 70 oz. Saline purges—a blister.* *12th.* Pain of chest easier—breathing oppressed. *Tinct. digital.—anodyne at bed-time.* *13th.* Much the same, but, in the afternoon, there came on pain in the region of the liver, increased on pressure. *V. S. ad ℥xxiv.—castor oil—turpentine enema—anodyne.* *14th.* Delirious—respiration oppressed—pulse 144, feeble. Died at 5, p. m.

*Sectio cadaveris, fifty hours after death.*—In consequence of the heat of the weather, putrefaction had made some progress. There was some pus in the anterior mediastinum, and about a pint of greyish muco-purulent matter in the right cavity of the pleura, with a little bloody extravasation into the left. The wound had adhered by the first intention, but was partially opened up by putrefaction. The carotid artery appeared to be quite sound in the neck—small clots had formed above and below the ligature, and the artery was puckered from the recent deposition of lymph. Its inner coats were divided, its external entire, but below the ligature the inner coat was of a vermilion tint, as was that of the thoracic aorta. The aortic arch between the heart, and the giving off of the left carotid, was healthy. The temporal and other branches of the carotid in the head "had degenerated into dilated tubes of great thinness and transparency," and had become elongated, contorted, and convoluted on themselves, so as to form, by this species of doubling, the tumours which constituted this singular disease. Where the thinning of the branches began, or whether the internal branches of the carotid had suffered also, giving rise to the epileptic fits to which he had recently become subject, Dr. M. was prevented from ascertaining.

Dr. Maclachlan thinks, and so do we, that there is an obvious difference between this species of pulsating tumour, and the aneurism by anastomosis described, by John Bell, as "a congeries of active arteries, absorbing veins, and intermediate cells." Two cases are given by Pelletan in his Clinique Chirurgicale, precisely tallying with this. One patient was a girl of 18, on whom compression was tried, but she could not bear it. He then tied the temporal artery, and with good effect, but the patient died of "indigestion." Upon dissection, the tumour was found to consist of tortuous and dilated arteries. Boyer, Pelletan, and others, are of opinion, that the disease is congenital, but without sufficient grounds. Dr. M. himself opened the temporal artery in the first instance, and, at that time, there was no trace of enlargement. We now come to the question of treatment. In all the cases of the affection which have been published, compression has failed, on account of the pain which it induced; and, when we consider the anatomy of the parts, we might be led to expect as much. Ligature of the temporal artery has been tried, but, although it gave a temporary relief, it does not seem ever to have effected a cure. This, too, might have been expected; for, although the disease consists, in the main, of the convolutions of one trunk, the temporal, still the anastomoses between it and its fellow of the opposite side, as well as with the occipital and even the supra-orbital artery, must always be enough, and more than enough, to allow of the *free* ingress of the blood into the tumour. If this reasoning applies, and we believe that it does so, to the tying of the vessel which *immediately* feeds the tumour, must it not, à fortiori, apply to the ligature of that great trunk which does *not* immediately feed it, we mean the common carotid? You tie the carotid, you expect to obliterate its dilated temporal branch, and why? Is it because a similar operation on the femoral artery will cure a popliteal aneurism? The analogy is not a fair one. Between the point of the ligature and the sac, there come off in the femoral no branches of any consequence. One or two of the articular arteries may arise just above the sac, but they are small, and, at any rate, anastomose with the tibials below, so as not to interrupt the remora in the sac itself. The new circulation is carried on principally by the branches of the profunda and common femoral, (we mean its long external descending ramus,) and *not by the vessels of the artery tied.*

With the carotid it is exactly the reverse. First, there is the division into internal and external, and, prior to this last terminating in the internal maxillary and temporal, there are *eight branches given off*, all of them having communications, more or less free, with each other. This being the case, we put it boldly to any man of common sense, whether he can reasonably expect to obliterate an enlargement of the *terminal* branch of a vessel, whilst a full and free circulation *must* necessarily be kept up between that enlargement and the ligature. The consequence of tying the common carotid is simply this, that the vessel is obliterated nearly as high as its division into the external and internal, but not one jot higher, because, above this, the circulation is brought *into* these vessels by their anastomosis with the inferior thyroid of the same side, and the thyroid and other branches of the opposite, as well as by the free inosculations of the internal carotid. In fact, the man who imagines he can plug up the temporal artery by an operation on the common carotid, might just as fondly attempt to obliterate the vessel of the great toe, by tying the artery at the groin!

So much for what the operation has *not* accomplished, but let us look a little at what it *has*. In the case detailed by Dr. Maclachlan, there cannot be a doubt that it was the immediate cause of the patient's death—in the case which we are now to detail, there can be as little doubt that it led ultimately to the same end.

J. Nowlan, æt. 22, was admitted into Panton-square, with a pulsating swelling on the left parietal bone, in the situation of the posterior temporal artery. It could be emptied by pressure, but a large communicating branch passed across the vertex from the opposite temple, and contributed to supply it with blood. The cranium beneath appeared to be very considerably absorbed, and the integument above was stated, in the Lancet, to be blue and ready to ulcerate, but the latter statement we have heard denied. The temporal artery had been secured, at least it was so supposed, by Mr. Babington, but with no effect. The day after the man's admission, the common carotid artery was tied by Mr. Wardrop, in order, as the reporter tells us "to completely arrest from almost every channel, the supply of blood to this rapidly increasing vascular tumour." Much difficulty was experienced in getting the needle round the artery, and a free bleeding took place (so goes the report) from *two large thyroideal veins*. The pulsation ceased, but the tumour did not collapse, and next day a thrill was again perceptible. On the fifth day, there was much fever, and he was bled to syncope—on the 7th, hæmorrhage, partly venous and partly arterial, occurred to a considerable extent, and he was again bled to fainting. On the 9th day, he was once more largely bled, venous hæmorrhage had occurred daily since the operation, the wound was not disposed to unite, and the tumour distinctly pulsated. *11th day.* Pain in the left eye-ball and orbit, with deafness, drowsiness, and mental disturbance, which increased on the 12th almost to coma, with contracted pupil. On the *13th day*, he was better, but the eye-ball was found to be greatly protruding, which was attributed, by Messrs. Lawrence and Wardrop, to a powerful compression on the brain, exercised by an aneurismatic state of the vessels of the dura mater, communicating through the skull with the tumour on the head! This was certainly an odd notion enough, and turned out to be any thing but the true one. The protrusion in the eye-ball, however, continued to increase—the sclerotica sloughed, and the humours were discharged. The ligature came away on the 25th, and the wound quickly healed.

On the 26th October, the case was brought forward by Mr. Duncan, the very intelligent house-surgeon at Panton-square, in the Westminster Medical Society. After detailing the case, he requested the opinions of the members on the treatment to be adopted, for, at this time, the tumour was very little better than before the performance of the operation. It would not be difficult to raise a laugh, were we to record many of the opinions as to the nature of the disease expressed by gentlemen on that occasion. Mr. Mayo proposed excision of the tumour. Dr. J. Johnson recommended circumvallating it by a circular incision, and tying the vessels which fed it directly, as well as those which supplied it by anastomosis. Nothing further was heard about the case until the 4th January, when the poor fellow was admitted into the Middlesex Hospital, with lumbar abscess, and altogether in a most miserable state. On the 21st he died. The tumour, at this time, was large and pulsated very strongly.

On dissection,* the posterior temporal artery was found to divide into two branches, one running, as usual, beneath the skin, the other perforating the tendon of the occipito-frontalis, and then, considerably enlarged, twisting and turning and coiling upon itself, looking, when injected, as like a varicose vein, as it is possible for a vessel which has no valves, to resemble one which has them. On examining the neck, the common carotid was obliterated, and converted into a cellular chord just up to its bifurcation, whilst the jugular vein was in nearly the same state of degeneration from the site of the ligature up to the division of the carotids, from which to the base of the brain it was plugged with coagulum. There was much pus at the basis cranii, and around the commissura tractuum opticorum; it lay between the pia mater and arachnoid, and was continued within these membranes down the whole length of the spinal chord. The cranium was somewhat grooved beneath the tumour, just as it would be beneath a powerful muscle, but the pericranium was entire, and the inner surface of the bone was *perfectly smooth and equal.*

We have thus given an abstract* of this very interesting case, and it seems to us entirely to confirm what we observed upon Dr. Maclachlan's. It establishes most completely the inutility of the ligature upon the carotid, for that vessel was plugged up no higher than (indeed not so high as) its bifurcation. This is a fact of some importance, for, although we cannot well be accused of belonging to that IGNAVUM PECUS, who are eternally prating about "the wisdom of our ancestors," and entertain a most conscientious horror of every thing which was not known to pathologists in the good old days of Noah, still we do think that if any thing is carried to excess at the present time it is the rage for tying carotid arteries. The operations on this vessel which have been performed of late by Mr. Wardrop, have been most unfortunate, and sorry as we should be, to attach blame to any man who endeavours to establish a principle in surgery, we would yet intreat Mr. Wardrop for his

* The principal facts stated here were given by us upon the cover of the last Fasciculus. We thought it better, however, to repeat them, that the case might not be incomplete.

* The account of the case is culled from the LANCET; the dissection is recorded in the 9th Number of the MEDICAL GAZETTE, but we had also an opportunity of inspecting the parts ourselves.

own sake, and for the sake of science, to pause awhile ere he repeats them. The only two proposals which appear to us in any way applicable to this pulsating tumour of the temporal artery, are those which were made by Mr. Mayo and Dr. Johnson, to wit, *excision*, and circumvallating the tumour by an *incision*. The second is certainly less formidable than the first, and would prove quite as effectual. If, as in Dr. Maclachlan's case, the disease be too extensive to admit of complete circumvallation, those branches which go *to*, and those which go *from* the tumour might still be tied, and that, from their increased size, without any great difficulty. This, after all, is in principle, only the operation daily performed upon a punctured artery, namely, the cutting off all influx of blood into the wounded part, by a ligature above and below. Some questions present themselves upon Mr. Wardrop's case, viz. the loss of the eye, and the obliteration of the jugular vein, but our limits will not allow us to consider them fully. We think, however, from the difficulty experienced during the operation—from the venous bleeding at the time, and from that which occurred afterwards, that the needle was unfortunately pushed through the coats of the vein, and part of it, at any rate, tied with the artery. Whether the ligature of both had any effect upon the eye, is more than we can pretend to say.

---

### 6. BARTHOLOMEW'S HOSPITAL.

### SCIATICA CURED BY MOXA.

In the seventh Number of the Gazette, there is an interesting case related from the practice of Mr. Earle. The patient was a courier, æt. 32, who began to be affected with sciatica—"racking sciatica"—two years previously, whilst travelling on the Continent. He had been cupped, leeched, and blistered—used hot baths and cold baths, mineral waters, &c. without the slightest benefit. When abroad, he had undergone acupuncturation three times, with some temporary benefit, but would not submit to the introduction of the needles any more, on account of the pain they occasioned. For fourteen months prior to his admission, his sufferings had been very severe, and his health was a good deal broken in consequence. He could lie only in the horizontal position, and referred the pain accurately to the course of the great sciatic nerve. Mr. Earle put him upon alterative doses of the blue-pill, with some relief to the pain, but not making progress under this plan, acupuncturation was tried, but only with slight effect. He was then ordered the subcarbonate of iron, ℈j. every four hours, the dose being gradually raised to ʒss. The medicine disagreed with his stomach, and was discontinued. Mr. Earle now applied a dozen moderate sized moxas along the thigh, and with the happiest effect, for the pain in the thigh never returned. It was necessary subsequently to re-apply the moxa on the loins in consequence of some pain there, which was not relieved by cupping and belladonna plaster. On the 1st January, he was made an out-patient, and continues so well, that he talks of resuming his old situation as courier.

We think the moxa is scarcely used enough by surgeons in this country. In some cases, it is decidedly preferable to cupping, on account of the manipulation and disturbance which the part must necessarily undergo during the latter process. In an inflamed joint, for instance, we have again and again, seen cupping ordered, the surgeon not considering that the pressure of the glasses and scarificator, and the disturbance done to the joint, must inevitably do, at least as much mischief, as the local depletion can do good. As far as we have seen, however, the moxa is not so applicable to the acute stages of inflammation, as to the more chronic forms of sciatica—old sprains—stiff joints after rheumatism, and cases of that description.

---

### 7. GUY'S HOSPITAL.

### HYDROCELE TREATED ON BARON LARREY'S PLAN.*

The patient was a robust blacksmith, æt. 32, the subject of a large hydrocele, which had appeared gradually after a blow, inflicted twelve months previously.

---

* London Med. Gazette, No. 8.

The tumour was tapped by Mr. Key, on the 8th December, and 22 ounces of fluid drawn off. A common gum-elastic catheter was then introduced through the canula into the tunica vaginalis, secured by adhesive plaster, and the canula withdrawn. Severe pain in the testicle and cord ensued, effusion of a gelatinous consistence took place, and, on the 11th, the instrument was removed, but a fresh secretion of serum into the tunica vaginalis being the consequence, the catheter was again introduced on the 20th. On the 23d, decided symptoms of inflammation had taken place, and it was again withdrawn. Adhesive inflammation now came on, and on the 21st January, the patient was discharged, a trifling induration in the tunica vaginalis only remaining.

As an experiment, this case is satisfactory enough, but certainly the catheter will never supersede the ordinary method of injection for the cure of hydrocele. The common operation is over in a few minutes, is productive of but little pain, and is exceedingly successful. This, on the contrary, is tedious, painful, and the result any thing but certain. There are, however, some cases where injection has failed, and where the surgeon is obliged to resort either to incision, or the caustic potash, both which operations are extremely severe, and not entirely devoid of danger. It is in these cases, we think, that Baron Larrey's operation is deserving of a trial, as it is much more mild than either of the above, and not, by any means, so likely to induce constitutional disturbance.

Mr. Key has, also, treated a case of "house-maid's knee" in the same manner, and with the same success. The trocar and canula were pushed into the most depending part of the tumour, three ounces of fluid, having a reddish-yellow colour and partly coagulating by heat, drawn off, the gum-elastic catheter introduced into the cyst, and a roller applied around the joint, which was kept cool by evaporating lotions. Much inflammation was induced, and, on the 12th day, the catheter was removed, when pressure and the emp. ammon. c. hydrarg. completed the cure.

The treatment is only applicable to bursæ which contain fluid. In such cases, the evacuation of the fluid by a puncture, and subsequent active blistering, will not unfrequently effect a cure. A very short time ago, a woman was admitted into St. George's Hospital, with a swelling, the size of a pigeon's egg, in the right ham. It had no pulsation, had an undulatory fluctuating feel, and was not very painful upon pressure. The patient stated that it had been coming on for eight years, had been once entirely removed by mercurial ointment, but subsequently returned, and was attended with such pain as to completely lame her. Mr. Brodie imagining it to be an enlarged bursa, punctured it with a fine needle, when there dribbled out about an ounce of yellowish fluid, of an oily consistence, which entirely coagulated upon heat. A blister was applied, and kept open by the savine ointment, and when, we saw the patient last, the tumour had almost entirely disappeared.

If, however, the bursal sac, instead of containing fluid, has become converted, as happens in a great many cases, into a solid tumour, with hardly any cavity in its centre, it is clear that neither the introduction of a catheter, nor incision, can be of any service. In such a case the sac must be dissected out, and it is really surprising how little disturbance such an operation will cause in a tolerably healthy constitution. We lately saw an instance where this operation was performed by Mr. Rose at St. George's Hospital, and the inflammation which followed was exceedingly trifling. At the same time, it cannot be denied that bad, and even fatal, consequences will occasionally follow the excision of the bursa, and that, sometimes, without the surgeon being able to detect the why or wherefore. Not very long ago we recorded a case of this kind, which happened to M. Velpeau, at the Hôpital de Perfectionnement.

---

### 8. ST. GEORGE'S HOSPITAL.

#### PECULIAR AFFECTION OF THE THIGH.

Two cases of a peculiar affection of the thigh, dependent on rheumatic inflammation, and terminating in ulceration of the cartilages, are related in the 8th No. of the Medical Gazette. The symptoms presented by both patients bear a very close resemblance, and the affection appears to be an exceedingly severe, and even dangerous one.

In either case the swelling came on suddenly, and was marked by some peculiar characters. It extended from the upper part of the thigh affected, over the knee, for some little distance down the leg—it was not circumscribed, but still its principal seat was evidently in the lower part of the thigh and knee, above and below which it gradually passed away—it was tense—elastic—exquisitely painful on the slightest pressure, and of a glossy, marbled white, bearing a very striking resemblance to the description of phlegmatia dolens. The patient could not support the least motion of the knee, but the pain, though partly in the joint, appeared to be principally in the textures around it. These, with considerable irritability and feverishness, were the symptoms presented, upon their admission, by the patients, both of whom had been much exposed to damp and cold, the first being a common prostitute—the second, a washerwoman.

We shall not follow up the details of the individual cases; suffice it to say that, in the first, which was under the care of Mr. Brodie, leeches frequently repeated, with calomel and opium, so as to affect the mouth, were employed, and with temporary benefit. Symptoms, however, of ulceration of the cartilages of the knee-joint supervened, and were attended with the most intense suffering. Leeches, blisters, fomentations and plasters of belladonna, and, lastly, a caustic issue were employed, but with no permanent good effect. Ulcers formed over the sacrum, which somewhat relieved the pain in the knee, but the left shoulder now became affected with excruciating pain, and a bilious diarrhœa came on, and carried off the unfortunate patient. On dissection, the cartilages of the knee-joint were found extensively ulcerated, and the periosteum of the femur, though not perceptibly thickened, was easily separable from the bone, which was preternaturally vascular.

The only remedy which had even a temporary effect in this case, was the calomel and opium, and Mr. Brodie, in his clinical lecture, stated that he had met with symptoms precisely similar, in a page in a nobleman's family, which were successfully treated by the above remedies. The case which we noticed in our first fasciculus, (page 450) appears to us to have been the same, or nearly the same, affection, save that, there, the inflammation had not attacked the joint, and had taken on the chronic, instead of the acute form. The second case we do not deem it necessary to relate at length here. It is still under treatment by Mr. Keate, and the reporter states, that the woman "bids fair, from the similarity of symptoms, of constitution, and even of *personal appearance*, to fall into the same state as the other unfortunate patient did before her."

---

### 9. ST. THOMAS'S HOSPITAL.

#### FURIOUS DELIRIUM TREATED BY MERCURY.

A young man, lately from the country, but, for the last six weeks, in Barclay's brewhouse, was received into St. Thomas's Hospital (having been three days ill) with *furious* delirium, constant jactitation, spitting of viscid saliva, flushed face, hot dry skin—answers questions in a loud, hurried, and anxious manner—pulse 60, full, and firm. Was bled, and fainted when sixteen ounces were abstracted—after which, he was more tranquil, but seemed much exhausted. Ten grains of calomel every four hours. Towards evening, (5th Jan.) he became more anxious, and ultimately furious. Twenty-four leeches to the temples, with another interval of quietude. At 5 o'clock in the morning of the 6th, the delirium again returned. Eighteen ounces of blood were drawn, and produced syncope, after which, he slept, and was then more composed. But he still spoke in a hurried manner, and the pulse was 150, easily compressible. The calomel in the above-mentioned doses, was still taken regularly. *Had three dark, offensive, and liquid motions. Evening of the 6th.* Is more furious and restless—flushed face—constant spitting—pulse quick, not firm. Leeches to the temples—blister to the head. The calomel every three hours. The mouth became sore at 8 o'clock in the morning of the 7th, and "*directly the gums were decidedly affected*—he became tranquil." The pulse fell to 80, and the patient rapidly recovered.—MEDICAL GAZETTE.

*Remarks.* We have no fault to find with the practice here pursued by Dr. Elliotson; but we have some doubts as to the disease being *pure* phrenitis. We have seen several instances of this kind, where furious delirium was produced by intestinal irritation, and we think the above case was one where the said irritation bore a considerable share in the production of the symptoms. Probably too, the young man had been making free with "Barclay's ENTIRE," and a dash of delirium tremens might have entered into the composition of the malady. The pulse at 60—the black stools—the varying state of the cerebral affection—the syncope from small detractions of blood, and the previous history of the case, incline us to this opinion, but we may be wrong.

---

### MEDICAL JURISPRUDENCE—CORONER.

On Saturday, the 2d February, Mr. Barrett Marshall read a learned and eloquent paper on Medical Jurisprudence, more especially in relation to wounds and to Coroner's inquests. There was no professional discussion, but a stormy debate ensued, respecting the propriety, or rather the necessity, of the office of Coroner being vested in a medical man. This subject has lately been canvassed in one of the weekly journals, without much benefit, we fear, on account of the intemperate and passionate manner in which the question has been approached. For our own parts, we never wish to see the medical character mixed up with the *administration* of the laws of the land. The only *power* which the physician or surgeon should aspire to, is KNOWLEDGE. It was maintained by some of the members, that the duty of the Coroner required no legal knowledge. This we apprehend to be a mistake. In the article CORONER, in Rees' Cyclopœdia, it is stated that his "authority is judicial and ministerial"—that "he is to commit those to prison, who are found guilty, by his inquest, of murder or other homicide;"—"he must, also, inquire concerning their lands, goods, and chattels, which are forfeited." "Another branch of his office is to inquire concerning shipwrecks—and find out who is in possession of the goods." In short, we conceive that the Coroner's office is a judicial situation, which is by no means adapted to the medical character. But we admit—it cannot be denied, that, in almost all cases of Coroner's inquests, the KNOWLEDGE of the medical man is required. This knowledge may surely be put in requisition, without any necessity for the surgeon being himself a Coroner. The whole of the objections now urged against Coroner's inquests might be easily removed, by the appointment of a well-informed surgeon to assist the Coroner, not only by careful examination of the body, but by interrogating the witnesses on such points as fall within his department. It should be, and we believe it is, in the power of the Coroner, to summon, in difficult cases, several medical men, in order to have the benefit of their united knowledge. A great outcry is made, as to the necessity of minute medical knowledge in the Coroner; but we apprehend that, before we urge the legislature to appoint medical men to the Coroner's office, we should first see that medical men are themselves properly qualified. Minute medico-legal knowledge is necessary, and yet medical jurisprudence forms no part of the system of medical education (if system it can be called) in this country! Let us lay the axe to the root of the tree—or rather of the evil. The whole profession should unite in one general petition to the legislature, for a regulation of medical instruction. One uniform system of medical education should be instituted, and enforced by law—and then let individuals practise such branches afterwards as their genius suited, or their circumstances and inclinations rendered eligible.

We shall immediately proceed to the subject of medical education generally, and hope to shew, in a calm and argumentative manner, the baneful effects of the present system—or rather systems, in this country. We shall also endeavour to direct the profession to the only legitimate path of reform—a parliamentary investigation.

